# Impact of work-family support on job burnout among primary health workers and the mediating role of career identity: A cross-sectional study

**DOI:** 10.3389/fpubh.2023.1115792

**Published:** 2023-02-24

**Authors:** Diling Yang, Guixia Fang, Danmin Fu, Mengyuan Hong, Haoyu Wang, Yuqing Chen, Qinglian Ma, Jinxia Yang

**Affiliations:** ^1^Department of Health Service Management, School of Health Service Management, Anhui Medical University, Hefei, China; ^2^School of Marxism, Anhui Medical University, Hefei, China

**Keywords:** primary health workers, work-family support, career identity, job burnout, mediation effect

## Abstract

**Objective:**

In China, medical workers in the primary sector shoulder the task of providing people with the basic medical and public health services, and are the “gatekeepers” of the public health. This study aims to analyze the current situation of job burnout among primary health workers of China, and shed light on the effect of work-family support and career identity on job burnout among them and their relationships. This may provide a new perspective for primary health care institutions and health administrative departments so as to formulate policies to “attract, retain and stabilize” primary health workers.

**Methods:**

A multi-stage sampling method was adopted to select 8,135 primary health workers from 320 primary health care institutions in a province of central China. A descriptive statistical analysis, univariate analysis, Pearson correlation analysis, and mediation effect analysis were applied to analyze the effects of work-family support and career identity on job burnout among primary health workers as well as the mediating role of career identity.

**Results:**

Among 8,135 primary health workers, 4,911 (60.4%) participants had mild to moderate levels of job burnout, 181 (2.2%) participants had severe job burnout, and the burnout detection rate is 62.6%. Work-family support was negatively correlated with job burnout (*r* = −0.46, *p* < 0.01) and positively correlated with career identity (*r* = 0.42, *p* < 0.01). Work-family support (β = −0.346, *p* < 0.01) and career identity (β = −0.574, *p* < 0.01) were negative predictors of job burnout respectively. In addition, career identity had a mediating effect between work-family support and job burnout, with the mediating effect contributing 33.7% to the overall effect.

**Conclusions:**

The findings of this study demonstrate that work-family support is a protective factor against job burnout in primary health workers and reveal that career identity is a critical mediating mechanism linking work-family support to burnout. We propose to reduce job burnout by strengthening work-family support (especially work support), enhancing career identity, increasing the number of primary health workers and reducing the workload of existing incumbents, which can provide important practical implications for the future prevention and intervention programs.

## 1. Introduction

### 1.1. The importance of primary health workers in maintaining the health of the residents

Stabilizing the primary health workforce and improving the capacity of primary health services have always been the focus of China's medical and health system reform (1), as well as an important strategy to promote the formation of a hierarchical diagnosis and treatment pattern that can improve the efficiency of health resource utilization. In China, primary health workers bear the heavy responsibility of providing basic medical and basic public health services for the people. They are also the bottom of the network of the entire health service system, and the “gatekeepers” of the public's health (2). However, according to *China Health Statistics Yearbook 2021*, the number of primary health workers per 1,000 resident population in 2020 is only 3.07 (3), far short from the requirement in the *Outline of Medical and Health Service System Planning (2015–2020)*, which states that China should have had at least 3.5 primary health workers for every 1,000-resident population by 2020 (4). In addition, it is difficult to effectively replenish the primary health workers due to the deficiency of the current personnel recruitment system, unattractive remuneration package and career prospect (5). At present, hierarchical diagnosis and treatment system is being gradually promoted; the coverage of family doctor who provide contracted services for residents is increasing; the content of service packages provided by the national basic public health service program has increased from 41 items of nine categories in 2009 to 55 items of 14 categories in 2017 (5); and the COVID-19 epidemic is in the stage of regular management. All these circumstances have imposed new requirements on the quantity and quality of primary health workers. The continuous increase in workload makes medical workers in the primary sector more prone to job burnout, especially in poor areas (6, 7).

### 1.2. Job burnout and its hazards among primary health workers

Job burnout is defined as a state of physical and emotional exhaustion caused by excessive and sustained levels of work-related stress, characterized by emotional exhaustion (EE), depersonalization (DP), and low personal accomplishment (LPA) (8, 9). Some studies have shown that high burnout among health care workers not only leads to mental and physical health problems such as depression, suicide, sleep disorders, and cardiovascular disease, but also results in poor quality of healthcare and reduced work productivity (10, 11). Within health care organizations, burnout is related to high job turnover and early retirement (12). Wu et al. examined burnout among rural physicians in Jilin Province and found a 65.13% prevalence (13). Liu (14) investigated 650 medical workers in community healthcare centers in Fengtai District and found that the overall detection rate of burnout was 61.53%. The COVID-19 pandemic presents new social and work-related factors that increase the risk of burnout for health care workers (15, 16). Therefore, under the new situation, it has become an urgent concern and problem to effectively reduce the burnout of primary health workers, summon up their work enthusiasm and stabilize the primary health care teams.

### 1.3. Work-family support and job burnout

Conservation of resources theory suggests that a lack of work resources is liable burnout (17). Social support is also a resource, and there has been a great deal of research showing that social support is negatively related to burnout levels (18–20). However, the existing social support scales only measure the social support that individuals receive in the work and family domain separately, lacking an examination from a holistic perspective. Li and Zhao (21) argued that work-family support should be a two-way street, so they define work-family support as “the support that employees receive from both the work and family domains during the work process that achieves work-family balance.” Karagöl and Kaya (19) assessed burnout, hopelessness, and social support among health care workers during the COVID-19 pandemic and found that their own sense of control over their careers and social support from others were the two factors addressing job burnout, and that family support was the only support addressing the three sub-dimensions of burnout (EE, DP, and LPA) and hopelessness. Wang et al. (20) concluded that perceived social support, especially family support, plays a significant moderating role between emotional exhaustion and subjective wellbeing, and that improving perceived social support could reduce job burnout. Therefore, work-family support should be considered as an influencing factor of job burnout, and we propose hypothesis 1: Work-family support has a significant negative effect on job burnout among primary health workers.

### 1.4. Career identity and job burnout

Career identity is concerned with the social meaning and value of the work one engages in Wang et al. (22). According to Ashforth and Humphrey (23), there is less contradiction between expressive behavior and emotional experience amongst employees with higher career identity, and the positive emotions generated by career identity enables employees to adapt themselves to the display rules without emotional exhaustion. Onyett et al. (24) concluded that there was a significant negative correlation between career identity and two dimensions (emotional exhaustion and depersonalization) of job burnout. In a study of 53,236 Chinese general practitioners, Zhang et al. (6) found that career identity was negatively associated with job burnout and turnover intention. The higher the level of career identity is, the lower the level of burnout and tendency to quit will be. Therefore, career identity should be considered as an indispensable influencing factor of job burnout, and we proposed hypothesis 2: Career identity has a significant negative effect on job burnout among primary health workers.

### 1.5. Work-family support, career identity, and job burnout

Although previous studies have shown that medical workers' burnout is related to age, title, education, and working hours, as well as closely related to their own emotional state, career identity, and social support (25, 26), the underlying mechanisms behind these associations are unclear and need to be demonstrated through different research designs. Support, assistance, feedback and appreciation from colleagues, supervisors and families would create a supportive work environment for primary health workers which is conducive to the satisfaction, self-esteem, security and career identity gained from their work, which in turn plays an important role in alleviating job burnout (27). Thus, work-family support may influence job burnout through career identity, and we propose hypothesis 3: Career identity plays a mediating role in the relationship between work-family support and burnout levels of primary health workers.

At present, academics are more likely to study the influencing factors of job burnout from the perspectives of demographic factors and job characteristics (28), but other potential factors (e.g., career identity, work-family support) are studied in isolation, and few studies have combined them together. We assume that there is not only a direct relation between these variables and job burnout, but an indirect mediating effect. Further research is necessary to validate these hypotheses and elucidate the interactions between these parameters. In addition, in terms of research subjects, previous studies have mainly involved teachers (29), nurses (30), social workers (31) etc., but little attention has been paid to the job burnout of primary health workers. Therefore, this study, which focuses on the job burnout of primary health workers who have dual attributes of service and emotional labor, aims to clarify the relationship between work-family support, career identity and job burnout. This study attempts to probe into the influencing mechanism of job burnout of primary health workers and find effective paths to reduce burnout. The evidence may provide a different perspective for primary health care institutions and health administrative departments to develop policies and interventions to “attract, retain, and stabilize” primary health care workers.

## 2. Methods

### 2.1. Participants and procedures

Community healthcare centers (CHCs) and township health centers (THCs) are the main institutions providing basic medical and public health services to urban and rural residents. In this study, multi-stage sampling was used to select participants. Firstly, a province in central China was selected (there are 16 prefecture-level cities in this sample province). Secondly, through typical sampling, 10 township health centers and 10 community healthcare centers were selected from each prefecture-level city, with a total of 320 primary health care institutions. Finally, from March to May 2022, all primary health workers (including general practitioners, nurses, public health physicians, pharmacists, etc.) who met the inclusion criteria in the sample primary health care institutions were surveyed by cluster sampling. Inclusion criteria of participants was: (1) staff who had engaged in primary health services for 1 year or more; (2) informed consent and voluntary to participate in this study.

Once a contact was established with the survey sites, electronic online questionnaires were distributed to them through “WeChat Questionnaire Star” (WeChat is a widely used social media app in China and Questionnaire Star is a mini program within WeChat ecosystem) with the cooperation of the chiefs of the primary health section of each municipal health administration department. The survey was conducted anonymously among all medical workers in primary health care institutions who met the inclusion criteria. A total of 8,339 questionnaires were collected, and duplicate questionnaires were discarded after IP checking. After completeness and standardization of the completed questionnaires were verified (54 repeated filling and 150 missing key information), 8,135 valid questionnaires were finally determined, with an effective recovery rate of 97.5%. All the procedures complied with the ethical standards of the Anhui Medical University Committee (No. 83220442).

### 2.2. Measurements

#### 2.2.1. Demographic characteristics

Sociodemographic information and job characteristics of primary health workers were collected according to the needs of the study, which included questions concerning gender, age, education level, professional title, years of experience, average annual income and average daily working hours.

#### 2.2.2. Work-family support evaluation scale

The work-family support questionnaire was developed by Li and Zhao et al. (21), which conforms to the Chinese cultural context. After exploratory factor analysis and validation factor analysis, two dimensions (work domain support and family domain support) with a total of 26 items were finalized. Work domain support (18 items) includes items such as “when I encounter pressure and resistance in my work, the work unit can always give encouragement and help,” “the work unit can provide us with good welfare benefits,” etc. Family domain support (eight items) includes items like “When there is a problem at work, my family can always take on it with me,” etc. Each item is scored by Likert 5-level scale, ranging from 1 (strongly disagree) to 5 (strongly agree). The total score of the Work-Family Support Scale ranges from 26 to 130, with higher scores indicating higher levels of work-family support among primary health workers. In this study, the Cronbach's alpha coefficient of the scale was 0.97.

#### 2.2.3. Career identity evaluation scale

With reference to the Nurse Professional Identity Scale translated and validated by Liu et al. (32) and the Medical Staff Professional Identity Scale developed by Wu (33), the career identity scale suitable for primary health workers was developed after discussion between the subject group and experts. The scale consists of 12 items, and each item is scored by Likert 5-level scale, ranging from 1 (strongly disagree) to 5 (strongly agree). The total score of the scale ranges from 12 to 60, and the higher the score, the stronger the career identity of the primary health workers. The Cronbach's α coefficient of the scale in this study was 0.90.

#### 2.2.4. Job burnout evaluation scale

This study adopted the Maslach Burnout Inventory-General Survey (MBI-GS) translated and revised by Li and Shi (34), with 15 items in total, including three dimensions of emotional exhaustion (EE, five items), depersonalization (DP, four items), and low personal accomplishment (LPA, six items). The scale applies a seven-level rating scale ranging from 0 (never) to 6 (every day). Higher scores on the EE and DP subscales indicate the higher degrees of job burnout, while LPA is inversely correlated with job burnout. The total score of the MBI-GS ranges from 0 to 90. The score of each dimension was the mean of its corresponding item score, and the composite score of burnout scale was (0.4^*^EE + 0.3^*^DP + 0.3^*^LPA). A composite score < 1.5 was judged as no burnout, 1.5–3.5 as mild to moderate level of burnout, and >3.5 as severe burnout (35). The Cronbach's α coefficient of the scale in this study was 0.88.

### 2.3. Data analysis

All analyses were performed in SPSS 26.0 with the significance level set to 0.05 (two-tailed). Measurement data were described using mean ± standard deviation (*X* ± S); count data were described using utilization or composition ratio (%). Statistical differences in outcome indicators (work-family support, career identity and job burnout) between two subgroups were compared using *t*-test, those differences between three or more subgroups were compared using ANOVA, and rank sum test was used when data did not satisfy the homogeneity of variance. Pearson's correlation analysis was used to test the relationship between work-family support, career identity and job burnout. The SPSS PROCESS macro 4.0 was used to test the mediating effects. Since the bootstrap methods have the most precise confidence intervals (*CI*) for indirect effects, the bootstrap estimation procedure (using a specified bootstrap sample of 5,000) was used to test the mediating effect of career identity in the relationship between work-family support and job burnout. The mediating effect was considered statistically significant when the bootstrap 95% CI did not include 0.

## 3. Results

### 3.1. Characteristics of the participants

Among the 8,135 primary health workers, 2,618 participants (32.2%) were male and 5,517 participants (67.8%) were female, mostly aged between 26 and 55 (89.9%), whose workplaces were mainly township health centers (69.8%), and whose education status was mainly college diploma (43.1%) and bachelor's degree (36.5%). The professional title of them was mainly junior (54.9%), the average annual income of them was mostly 50,000–100,000 CNY Yuan (41.9%), and the average daily working hours is above 8 h (52.3%). There were 3,043 participants (37.4%) who did not experience any burnout, 4,911 (60.4%) with mild to moderate level of burnout, and 181 (2.2%) with severe burnout (see Table 1 for further details).

**Table 1:** Univariate analysis and description of each scale (*n* = 8,135).

**Variables**	***N***	**Work-family support**		**Career identity**		**Job burnout**	

		***X*** ±**S**	***t*****/*****F*****/**χ^2^	***X*** ±**S**	***t*****/*****F*****/**χ^2^	***X*** ±**S**	***t*****/*****F*****/**χ^2^
Gender							
Male	2,618	99.91 ± 18.63	7.38^**^	51.46 ± 6.38	2.28^*^	27.45 ± 12.80	−1.11
Female	5,517	96.65 ± 18.59		51.12 ± 6.11		27.78 ± 12.58	
Workplace							
CHCs	2,454	98.03 ± 18.57	1.04	51.27 ± 6.22	0.41	27.56 ± 12.87	−0.54
THCs	5,678	97.56 ± 18.71		51.21 ± 6.17		27.73 ± 12.55	
Age group (years)							
≤ 25	509	95.66 ± 18.98	116.93^**^	48.99 ± 6.30	163.55^**^	31.37 ± 12.93	258.83^**^
26–35	2,319	94.98 ± 19.22		50.34 ± 6.45		30.44 ± 12.96	
36–45	2,608	97.73 ± 18.74		51.82 ± 6.06		26.75 ± 12.63	
46–55	2,386	100.17 ± 17.76		51.85 ± 5.91		25.48 ± 11.81	
≥56	313	102.17 ± 16.61		51.79 ± 5.83		25.63 ± 11.11	
Education level							
High school or below	1,636	101.12 ± 17.40	105.07^**^	51.15 ± 5.93	0.74	25.62 ± 11.83	74.82^**^
Junior college	3,504	97.98 ± 18.89		51.25 ± 6.28		27.55 ± 12.80	
College	2,972	95.54 ± 18.76		51.25 ± 6.25		28.93 ± 12.74	
Master or above	23	91.13 ± 19.09		50.61 ± 6.05		30.91 ± 12.69	
Professional title							
Senior	29	101.07 ± 17.77	6.27^**^	53.55 ± 4.48	124.17^**^	24.14 ± 10.80	6.76^**^
Vice-senior	346	97.14 ± 18.72		52.33 ± 6.10		26.75 ± 12.47	
Middle	2,440	96.65 ± 18.17		52.27 ± 5.77		26.80 ± 12.17	
Primary	4,467	97.81 ± 18.88		50.75 ± 6.25		28.30 ± 12.81	
None	853	100.25 ± 18.73		50.19 ± 6.73		27.42 ± 13.09	
Years of experience (years)							
≤ 3	873	97.04 ± 18.61	113.22^**^	49.60 ± 6.48	53.31^**^	30.04 ± 13.09	198.54^**^
4–10	1,802	95.06 ± 19.60		50.24 ± 6.46		30.31 ± 13.19	
11–20	2,029	96.13 ± 18.52		51.64 ± 5.99		27.68 ± 12.20	
≥21	3,431	100.19 ± 17.95		51.91 ± 5.96		25.69 ± 12.14	
Average annual income (CNY)							
< 30,000	1,257	97.01 ± 19.72	8.07^*^	50.28 ± 6.80	54.54^**^	28.42 ± 13.33	9.77^*^
30,000–50,000	3,059	98.13 ± 18.91		51.01 ± 6.17		27.53 ± 12.88	
60,000–10,000	3,410	97.40 ± 18.11		51.64 ± 5.95		27.70 ± 12.20	
≥110,000	409	99.13 ± 17.98		52.35 ± 6.12		26.28 ± 12.27	
Average daily working hours (hours)							
< 7	269	100.84 ± 18.35	29.26^**^	50.95 ± 6.34	19.29^**^	27.63 ± 12.66	18.16^**^
7–8	3,613	98.68 ± 18.21		50.98 ± 6.02		26.94 ± 12.24	
9–10	3,133	96.62 ± 18.84		51.38 ± 6.23		28.23 ± 12.85	
≥11	1,120	96.80 ± 19.44		51.65 ± 6.60		28.49 ± 13.24	

^*^p < 0.05.

^**^p < 0.01.

CHCs, Community Healthcare Centers; THCs, Township Health Centers.

### 3.2. Differences in scores across different groups

The results of the study showed that gender, age, education level, professional title, years of experience, average annual income and average daily working hours of primary health workers were independent influencing factors for work-family support (*p* < 0.05). Gender, age, professional title, years of experience, average annual income and average daily working hours were independent influencing factors for career identity (*p* < 0.05). Age, education level, professional title, years of experience, average annual income and average daily working hours of primary health workers were independent influencing factors of job burnout (*p* < 0.05; see Table 1 for further details).

### 3.3. Correlation between work-family support, career identity and job burnout

Inter-correlations, means, standard deviations and reliabilities of all variables were calculated to explore associations among different variables. The correlation analysis verified hypothesis 1 and hypothesis 2. The mean scores for each item of work-family support, career identity and job burnout for primary health workers were 3.76 ± 0.72, 4.27 ± 0.52, and 1.84 ± 0.84, respectively. Pearson correlation analysis revealed that work-family support was negatively correlated with job burnout (*r* = −0.46, *p* < 0.01) and positively correlated with career identity (*r* = 0.42, *p* < 0.01). Compared to work domain support, family domain support had a stronger effect on career identity. Career identity was negatively associated with job burnout (*r* = −0.48, *p* < 0.01). Work-family support and career identity were negatively correlated with all three dimensions of job burnout (see Table 2).

**Table 2:** Means, standard deviations, and correlations among all variable.

**Measures**	**Mean**	**SD**	**1**	**2**	**3**	**4**	**5**	**6**	**7**	**8**
Work-family support	3.76	0.72	(0.97)							
Work domain support	3.61	0.84	0.97^**^	(0.97)						
Family domain support	4.08	0.70	0.72^**^	0.52^**^	(0.94)					
Career identity	4.27	0.52	0.42^**^	0.36^**^	0.43^**^	(0.90)				
Job burnout	1.84	0.84	−0.46^**^	−0.41^**^	−0.41^**^	−0.48^**^	(0.88)			
EE	1.75	0.99	−0.47^**^	−0.46^**^	−0.32^**^	−0.30^**^	0.66^**^	(0.93)		
DP	1.10	1.02	−0.49^**^	−0.46^**^	−0.38^**^	−0.40^**^	0.75^**^	0.69^**^	(0.90)	
LPA	2.43	1.38	−0.18^**^	−0.13^**^	−0.25^**^	−0.36^**^	0.76^**^	0.07^**^	0.25^**^	(0.92)

1–3, work-family support and its dimensions; 4, career identity; 5–8, job burnout and its dimensions. Reliability coefficients (Cronbach' α) in parentheses along main diagonal.

EE, emotional exhaustion; DP, depersonalization; LPA, low personal accomplishment.

Analyses based on n = 8,135.

^**^p < 0.01.

### 3.4. Mediation effect analysis

**Figure 1:**
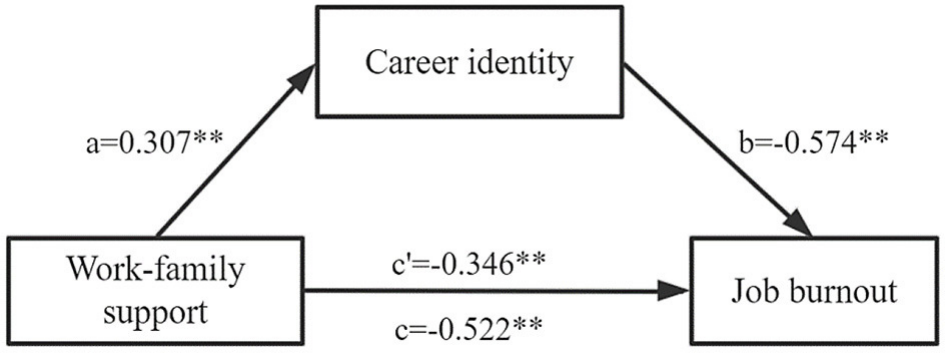
Schematic representation of the mediating effect of career identity between work-family support and burnout. *a, b*: mediating effect of intermediary variable career identity; *c*′: direct effects; *c*: total effects; ^**^p < 0.01.

The study hypothesized that career identity of primary health workers played a mediating role in the influence of work-family support on job burnout. The results of Table 3 show that the three models were statistically significant and the coefficients of each pathway had a significant effect (*p* < 0.001) when controlling the variables like the age, education level, professional title, working years, average annual income and average daily working hours of primary health care personnel. The results of the bootstrap mediated effect test showed that the bootstrap 95% *CI* for the indirect effect was (−0.191, −0.162), excluding 0. Since the signs of *ab* and *c*′ were the same sign, it indicated that there was a partial mediated effect, i.e., career identity played a partial mediating effect between work-family support and job burnout among primary health workers, confirming hypothesis 3. The contribution rate of the mediating effect to the total effect was: Effect *M* = *ab*/*c* =33.7% (see Table 4). A schematic representation of the mediating effect of career identity between work-family support and burnout is shown in Figure 1.

**Table 3:** Model testing of mediations.

**Model^a^**	**Outcome various**	**Predictors**	**Fitting index**		**Coefficient significance**		

			*R* ^2^	***F***	β	***t***	***p-*** **value**
1	Job burnout	Work-family support	0.226	338.54	−0.522	−44.94	< 0.001
2	Career identity	Work-family support	0.203	295.92	0.307	42.47	< 0.001
3	Job burnout	Work-family support	0.324	487.16	−0.346	−28.86	< 0.001
		Career identity			−0.574	−34.39	< 0.001

^a^Age, education level, professional title, years of experience, average annual income and average daily working hours of primary health workers were introduced into the model as control variables.

**Table 4:** Results of testing the mediating effect of career identity between work-family support and job burnout.

**Work-family support → Job burnout**	**Effect**	**Boot *SE***	**Boot*LLCI***	**Boot*ULCI***	**Effectiveness ratio (%)**
Total effects	−0.522	0.012	−0.545	−0.499	–
Direct effects	−0.346	0.012	−0.370	−0.322	66.3
Indirect effects	−0.176	0.008	−0.191	−0.162	33.7

## 4. Discussion

### 4.1. Variable description and differences

Health care professionals are generally considered to be one of the highest-risk groups experiencing burnout, given the emotional strain and stressful work environment of providing care to sick or dying patients (36). The results of this study showed that the burnout rate among primary health workers was 62.6%, a level between community physicians (61.53%) and rural physicians (65.13%) in previous studies (13, 14), and higher than the burnout rate among clinicians in the United States (35%−54%) (36–38). This is because in China, primary health care workers are responsible for both primary medical care and public health service, which means that in addition to their medical duties, they also need to undertake preventive care, patient rehabilitation and chronic disease management, health management and other tasks (39). The high-pressure, high-load work environment and highly stressful practices overtax the energy as well as the physical strength of primary health workers, leading to higher levels of burnout at work. In this study, particular attention is paid to primary health workers aged 25 years or below with a bachelor's degree or above, primary title, < 3 years of working experience, low annual income and average daily working hours >10 h. This study tries to help them cope with the contradiction between high expectations on reality and stressful dilemmas at work in a reasonable way. Young primary health workers with high education degree often have higher expectation of salary, higher expectation of work prospect and stronger desire for fulfilling self-worth. However, since they have just stepped into the society, they have to confront the fact of low salary, low professional title and long working hours. This will undoubtedly put them under greater pressure, and easily lead to psychological disparity and job burnout.

The mean item score of work-family support for primary health workers was 3.76 ± 0.72, which was at an upper middle level, with the level of work domain support slightly lower than that of family domain support. Further analysis found that the two items with the lowest scores were both in the workplace support domain (see Supplementary Table 1), namely “the work unit provides us with information about caring for the elderly and educating children” and “the work unit can provide us with good welfare benefits.” This suggests that primary health care institutions should increase material and welfare support for medical personnel, especially the support of the family, to reduce the impact of work on the family. By contrast, the two items with the highest scores were both in the family support domain, which was “My family always encourage me when I am tired from work” and “My family always do more housework when I am busy at work at a certain time.” This is particularly Chinese characteristic. In China, the health care professions are held to a higher ethical standard, advocating “sacrificing individual interests for public collective benefits, and sacrificing personal feelings for duty” (40). They are supposed to give priority to work when it happens to be a conflict between personal family and work. At this point, shifting family responsibilities to social support networks (e.g., parents, spouse) or seeking paid support becomes an unavoidable option, and other family members will share more family responsibilities to support the medical personnel's career. However, the emphasis on work responsibilities at the expense of sacrificing individual family needs of medical personnel is an over-exploitation of their professionalism and can easily lead to job burnout (41).

The items of career identity have an average score of 4.27 ± 0.52, which is at a high level. It shows that the primary health workers affirm the professional value and professional significance of grassroots work and are willing to dedicate themselves to the cause of grassroots health for life. Such high ideals and beliefs are the strong pillars that support them to stay at the grassroots and serve the needs of people's health. This study found that primary health workers with older age, higher professional titles, longer years of experience, higher annual income, and longer average daily working hours had a higher sense of career identity.

### 4.2. Work-family support affects job burnout

This study found that work-family support was negatively related to job burnout and its three dimensions, validating hypothesis 1, which is consistent with previous research findings (19, 42). Hobfoll's conservation of resources theory states that social support as a feature of the environment is an important resource to alleviate job burnout (43). When primary health care workers gain support from their work domain, such as primary health care institutions creating a good working environment, improving working conditions, and providing good welfare benefits, it makes them feel valued and experience a higher sense of security and belonging (44). When higher-level needs are fully satisfied, they will devote more time and energy to their work for the purpose of self-actualization, thus reducing the occurrence of job burnout. Furthermore, a study by Vignoli et al. (45) found that organizations were effective in mitigating work-family conflict when they provided employees with a range of family-friendly support policies. This also suggests that resources from the work domain not only save primary health workers the resources they need to manage work-family conflicts, but also bring potential resources from the family domain to help primary health workers cope with the demands of their work and become more focused on their tasks with enthusiasm. This study revealed that primary health workers who obtained more support from the family domain had lower levels of burnout, similar to the findings of Bakker and Shin (46, 47). When primary health workers are still burdened with higher intensity household chores after work or when their worries at work are not understood by their families, their level of psychological relief and relaxation will be even lower, negatively affecting their work engagement the next day.

### 4.3. The mediating role of career identity in work-family support and job burnout

This study not only creatively establishes the relationship between work-family support and job burnout but also reveals the effect path between them. The results demonstrate the mediating effect of career identity on the relationship between work-family support and burnout, confirming hypothesis 3.

The results show that work-family support for primary health workers positively predicts career identity. Primary health workers with higher levels of work domain support and family domain support are adept at using organizational and family support to break through difficulties as quickly as possible, even when they encounter difficulties at work, and in the process, their stress tolerance and sense of career identity are enhanced. This study further found that family domain support received by primary health workers contributed more to their career identity than work domain support from organizations, leaders, colleagues, etc. During the COVID-19 epidemic, many health care workers said that the understanding and support of their families was their inexhaustible motivation to stay on the front line of the fight against the epidemic (47).

The mediating effect test revealed that the career identity of primary health workers partially mediated the effect between work-family support and job burnout. When individuals have a higher level of career identity, they would devote more time, energy, and vitality to their work, and job dissatisfaction due to inadequate work support and family support may be reduced or even eliminated (48). Thus, it is clear that the hindering effect of work-family support on job burnout is partially achieved by strengthening career identity, and this pathway provides another perspective to explain the mechanism of job burnout.

### 4.4. Recommendations for alleviating job burnout among primary health workers

In response to the findings, this paper proposes countermeasures to reduce and alleviate job burnout among primary health workers in three aspects: enhancing work domain support, strengthening family domain support and improving career identity.

In terms of work domain support, according to Herzberg's two-factor theory, there are two groups of factors that influence people to be motivated to work: the first are hygienic factors and the second group are called motivating factors. Therefore, for one, primary health care institutions should make efforts to reduce workload, raise salary levels, promote teamwork, improve physical working conditions, and increase the number of primary health workers (49). For another, it is important to focus on motivating primary health workers, including a sense of job accomplishment, being acknowledged for their work, improving job evaluation system and increasing the level of participation in decision-making. For example, in the past, the proportion of medical personnel with intermediate and senior titles was smaller at the grassroots level, but now we can gradually increase their proportion, optimize the job settings, and smooth the promotion channels for grassroots talents (50).

In terms of family domain support, primary health workers and their family members can receive regular psychological counseling, stress management and family education (47). At the same time, primary health workers should increase communication with family members, especially when the workload is heavy. Good communication helps to obtain more understanding and support from families so that they can devote more energy to their work. In addition, given that work and family are two inseparable matters among employees, organizations need to cultivate a family-supportive working environment. Primary health care institutions and health administrative departments can consider implementing supportive policies and flexible work arrangements for primary health workers who suffer from family-to-work interference.

The career identity of primary health workers cannot be obtained without the acknowledgment and appreciation of their profession by society and the public (51). Therefore, the government should advocate that the whole society should care and respect medical personnel, especially elderly medical workers, rural doctors and grassroots health workers who are on the front line of fighting against epidemics, etc., so as to create a good societal atmosphere of respecting doctors and valuing health care cause (40). More importantly, the career identity of primary health workers can be improved by strengthening and implementing the primary care system, promoting the capacity of family doctor who provides contracted services, providing quality services to the contracted residents (52), enhancing the trust and satisfaction of residents in primary services (53), and improving the stickiness and dependence on primary health workers. In addition, school education should be strengthened to emphasize the career identity and ethics education of medical students in school. For example, career planning training should be conducted to encourage medical students to integrate their future prospect with the development of health care industry.

As last, it is also necessary to increase the number of primary health workers and to reduce the workload of existing incumbent staff. It is suggested that tuition-waived training of rural-oriented medical students should be carried out (54). Relevant administrative departments and medical schools should make full use of existing medical higher education resources for fresh high school graduates, using order-based training mode such as signing employment agreements with them before entering school and providing financial assistance for tuition during school. When they graduate, they are sent to work at the grassroots health care sector. In this way, a mass of primary health workers who are willing to take root in the grassroots and serve the people will be cultivated.

### 4.5. Limitations

This study has some limitations. First, the study was based on cross-sectional data without a follow-up survey of primary health workers, which may limit the ability to identify causal relationships between work-family support, career identity, and job burnout among primary health workers. Second, the current job burnout scale for primary health workers is not mature enough to be applied in China, and lacks clinically validated thresholds or critical values for burnout diagnosis, which may have subtle errors in the estimation of burnout rates. Finally, in the follow-up work, we can continue to conduct relevant studies on primary health workers in different provinces of China to make the results more representative.

## 5. Conclusion

Studying the job burnout of primary health workers and its influential factors as well as formulating measures to alleviate burnout are of vital importance to stabilize the primary health workforce, which would improve the quality and efficiency of services in primary health care institutions, and achieve health for all. Identifying solutions to alleviate job burnout among primary health workers is of interest to both researchers and practitioners alike (36, 44, 55). This study investigated the current status, influential factors, and internal correlates of work-family support, career identity, and job burnout among primary health workers. Primary health workers were found to have high career identity, and their perceived work-family support (especially from the work) was far from adequate and burnout rates were high. Moreover, findings of this study demonstrate that work-family support is a protective factor against job burnout in primary health workers and reveal that career identity is a critical mediating mechanism linking work-family support to burnout.

The findings of this study provide more empirical evidence for the two-way support relationship of work-family support. Furthermore, they contribute to a better understanding of the interactive mechanisms between work-family support and job burnout, and to clarifying the mediating role of career identity on the association, which provides the necessary theoretical basis for improving previous single-dimensional studies. In addition, we are concerned about the group of primary health workers working in rural areas and communities, and propose to reduce job burnout by strengthening work-family support (especially work support), enhancing career identity, increasing the number of primary health workers and reducing the workload of existing incumbents, which can provide references for primary health care institutions and health administrative departments to develop policies and intervention measures to attract and stabilize talents.

## Data availability statement

The original contributions presented in the study are included in the article/Supplementary material, further inquiries can be directed to the corresponding authors.

## Ethics statement

The studies involving human participants were reviewed and approved by Biomedical Ethics Committee of Anhui Medical University. Written informed consent for participation was not required for this study in accordance with the national legislation and the institutional requirements.

## Author contributions

DY drafted the manuscript. QM and JY framed the concept and designed the study. GF and DF conduct the data collection and material preparation. DY and MH performed the data analysis. HW and YC contributed to the production of some content of the draft. All authors read and approved the final manuscript.
