# Supplementary material for: Impact of work-family support on job burnout among primary health workers and the mediating role of career identity: A cross-sectional study
Source: Front Public Health. 2023 Feb 24;11:1115792. doi: 10.3389/fpubh.2023.1115792 (PMC9998699; doi:10.3389/fpubh.2023.1115792)
Supplement: Supplementary file 1 [file Table_1.DOCX]

***Supplementary Material***

**Impact of Work-Family Support on Job Burnout Among Primary Health Workers and The Mediating Role of Career Identity: A Cross-Sectional Study**

**Diling Yang^†^, Guixia Fang^†^, Danmin Fu, Mengyuan Hong, Haoyu Wang, Yuqing Chen, Qinglian Ma^*^, Jinxia Yang^*^**

*** Correspondence:**

Qinglian Ma: [qinglma@163.com](mailto:qinglma@163.com); Jinxia Yang: [yangjinxiaamu@163.com](mailto:yangjinxiaamu@163.com)

**^†^** These authors have contributed equally to this work and share first authorship.

# Supplementary Table 1

# Table 1: Average scores for each item on the Work-Family Support Scale

| **Items** | **X±S** |
| --- | --- |
| The work unit provides us with information about caring for the elderly and educating children | 3.34±1.16 |
| The work unit can provide good welfare benefits. | 3.49±1.10 |
| The leader will give us a holiday at an appropriate time to reunite with our families. | 3.53±1.09 |
| The work unit will often organize some activities that are beneficial to the physical and mental health of employees. | 3.54±1.07 |
| The work unit can provide a good employment environment, can care about us, and often talk to us. | 3.57±1,08 |
| Leaders will pay attention to the impact of work requirements on me and my family. | 3.57±1.01 |
| The work unit can give us flexible time to deal with work and family affairs. | 3.57±1.08 |
| I can easily talk to my leader about my family life. | 3.59±1.02 |
| When my work is affected by family or personal matters, the leader will understand me. | 3.60±1.01 |
| The work unit can meet our material and cultural needs and create a beautiful and comfortable working environment. | 3.61±1.06 |
| The leader usually cares about me. | 3.62±1.05 |
| When work mistakes are caused by family problems, the unit will understand them as appropriate. | 3.64±0.97 |
| My work achievements can always be affirmed and praised by the leaders. | 3.67±0.97 |
| When something needs me at home, the leader can let me go home early. | 3.67±0.99 |
| When my family encounters difficulties, the unit will give help. | 3.68±0.97 |
| When I encounter pressure and resistance in my work, the unit always gives encouragement and help. | 3.68±1.03 |
| When I have problems in my work, the leader will decide according to the situation, not just criticize. | 3.74±0.94 |
| My family is more interested in my work. | 3.89±0.91 |
| The unit can always give recognition to our work achievements. | 3.98±0.89 |
| I feel comfortable talking with my family about work. | 3.98±0.89 |
| When there are difficulties at work, my family always shares them with me. | 4.07±0.84 |
| When I have troubles at work, my family always understands my feelings. | 4.09±0.81 |
| After work, family members can always give some personal space. | 4.11±0.80 |
| My family always comforts me when I have problems at work. | 4.15±0.79 |
| My family always do more housework when I am busy at work at a certain time | 4.16±0.83 |
| My family always encourages me when I am tired from work. | 4.17±0.79 |
